# Changes in Compliance With Personal Preventive Measures and Mental Health Status Among Chinese Factory Workers During the COVID-19 Pandemic: An Observational Prospective Cohort Study

**DOI:** 10.3389/fpubh.2022.831456

**Published:** 2022-03-10

**Authors:** Jinqiu Yuan, Bolin Cao, Changhua Zhang, Paul Shing-fong Chan, Meiqi Xin, Yuan Fang, Yaoxi Chen, Dongfeng Huang, Lifang Li, Xujun Xuan, Gengpeng Zhang, Yihang Pan, Yulong He, Zixin Wang

**Affiliations:** ^1^Big Data Center, Precision Medicine Center, The Seventh Affiliated Hospital, Sun Yat-sen University, Shenzhen, China; ^2^Clinical Research Center, The Seventh Affiliated Hospital, Sun Yat-Sen University, Shenzhen, China; ^3^School of Media and Communication, Shenzhen University, Shenzhen, China; ^4^Center for Digestive Disease, The Seventh Affiliated Hospital, Sun Yat-sen University, Shenzhen, China; ^5^JC School of Public Health and Primary Care, Faculty of Medicine, The Chinese University of Hong Kong, Hong Kong SAR, China; ^6^Department of Health and Physical Education, The Education University of Hong Kong, Hong Kong SAR, China; ^7^Department of Rehabilitation Medicine, The Seventh Affiliated Hospital, Sun Yat-sen University, Shenzhen, China; ^8^Department of Andrology, The Seventh Affiliated Hospital, Sun Yat-sen University, Shenzhen, China; ^9^Department of Chinese Medicine, The Seventh Affiliated Hospital, Sun Yat-sen University, Shenzhen, China

**Keywords:** compliance with personal preventive measures, depressive symptoms, sleep quality, Chinese factory workers, observational prospective cohort study, COVID-19

## Abstract

Coronavirus Disease 2019 (COVID-19) vaccination together with good compliance with personal preventive measures may help eradicate the ongoing pandemic. This observational prospective cohort study investigated the changes in compliance with personal preventive measures, depressive symptoms, and sleep quality among factory workers within a 3-month follow-up period. A total of 663 workers were recruited by a stratified multi-stage cluster sampling in March 2020, and all of them completed a follow-up survey three months later. Multilevel logistic and linear regression models (level 1: factories; level 2: individual participants) were fitted. A significant decline was observed in consistent facemask wearing in workplace (from 98.0 to 90.3%, *P* < 0.001) and in other public spaces (from 97.1 to 94.4%, *P* = 0.02), sanitizing hands (from 70.9 to 48.0%, *P* < 0.001), household disinfection (from 47.7 to 37.9%, *P* < 0.001) and probable depression (from 14.9 to 1.5%, *P* < 0.001) over the follow-up period. A significant improvement in avoiding crowded places (from 69.8 to 77.4%, *P* = 0.002) and sleep quality (proportion of participants reporting poor sleep quality dropped from 3.9 to 1.2%, *P* = 0.002) was also observed. Efforts are needed to maintain compliance with personal preventive measures during the pandemic. Mental health problems were uncommon and likely to be one-off among Chinese factory workers.

## Introduction

Globally, the Coronavirus Disease 2019 (COVID-19) pandemic remains out of control. Factory workers is a sub-population of higher risk at COVID-19 infection than that of the general population, as many factories are crowded settings and it is hard for them to maintain physical distancing (1). COVID-19 outbreak in the workplace was reported in China and other countries (1–3). Moreover, most of the Chinese factory workers are young (4). Even infected with COVID-19, many of them may be asymptomatic and unaware of their infection; they may become a driving force of COVID-19 transmission in workplace and community (5, 6).

COVID-19 vaccination together with personal preventive measures may help to eradicate the ongoing pandemic. Use of facemask (7), hand hygiene (8), and physical distancing (e.g., avoiding social/meal gathering and avoiding crowded places) (9) are strongly advocated by the World Health Organization (WHO) and have been implemented worldwide (10, 11). The effectiveness of these personal preventive measures crucially relied on compliance by the public (12). Cross-sectional studies showed good compliance with personal preventive measures at the early phase of COVID-19 outbreak among Chinese factory workers (1, 13). However, there are concerns that when the number of daily confirmed COVID-19 cases began to decline, people's compliance with these personal preventive measures would start to drop as they believed the pandemic is under control. Modeling works showed that a decline in compliance with facemask wearing or physical distancing might result in new waves of COVID-19 outbreak in China (14). In fact, new waves of COVID-19 community outbreaks occurred in some Chinese cities since June 2020 (e.g., Beijing, Hong Kong, Urumqi, and Dalian) (15, 16).

COVID-19 resulted in an increase in mental health problems (17). Together with the unpredictability, uncertainty and fatal outcomes of COVID-19, measures used to control COVID-19 (e.g., lockdown and physical distancing) might lead to social isolation, loss of income, loneliness, and limited access to basic services (18). Several rapid cross-sectional studies reported that COVID-19 pandemic triggered mental health problems in the general public, such as stress, panic, depression, anxiety, and poor sleep quality (19–21). One of these studies reported a high prevalence of depression (19.6%), anxiety (12.3%), and poor sleep quality (14.9%) among Chinese workers who returned to work (22). Longitudinal studies in the U.K. observed an increase in anxiety and a decline in wellbeing during the pandemic as compared to the time before COVID-19 (23). However, it was unclear whether the impact of COVID-19 on mental health would be long-lasting.

It is important to identify which subgroups of factory workers may be at greater risk of non-compliance with personal preventive measures and poor mental health during the pandemic to inform health promotion and service planning. A cross-sectional study reported being female was associated with higher adoption of personal preventive measures (24). Regarding mental health status, longitudinal studies showed that depression and anxiety were greater in younger population, women, and those with pre-existing mental and physical conditions, and those in socio-economic adversity during the COVID-19 pandemic (23).

The stress-coping model was used as the theoretical framework in this study to understand the correlations between mental health and compliance with personal preventive measures. The stress-coping model has been used to understand the impact of life stress caused by COVID-19 on mental health and how mental health status would affect personal preventive behaviors during the COVID-19 pandemic (25, 26). The model suggests a bi-directional correlation. On one hand, the adoption of personal preventive measures constitutes a means of adaptive coping of stress caused by COVID-19, which would lead to better mental health. A Turkish study suggested that people who engaged in preventive behaviors against COVID-19 had better mental health during times of crisis (27). On the other hand, poor mental health status might result in maladaptive coping strategies, such as excessive hand washing or a disregard for preventive behaviors during COVID-19 (28). Previous studies hypothesized that characteristics associated with depression, such as low level of energy, a decreased focus, and greater hopelessness might result in disengagement in necessary health behaviors (29). Studies conducted in China and Japan showed that depressive symptoms might inhibit COVID-19 preventive behaviors (e.g., consistent facemask wearing and hand hygiene) (1, 30, 31).

To our knowledge, there was a dearth of longitudinal studies investigating changes in compliance with personal preventive measures and mental health among working population during the COVID-19 pandemic. To address these gaps, this observational prospective cohort study investigated the changes in compliance with personal preventive measures, depressive symptoms, and sleep quality among factory workers who resumed work within a 3-month follow-up period. We investigated the correlations between baseline characteristics (socio-demographics, compliance with preventive measures and mental health status) and compliance with personal preventive measures and mental health outcomes at Month 3. Our first hypothesis was that poorer mental health at baseline would be correlated with poorer compliance with personal preventive measures at Month 3. Our second hypothesis was that better compliance with personal preventive measures at baseline would be correlated with better mental health status at Month 3.

## Methods

### Study Design

**Figure 1:**
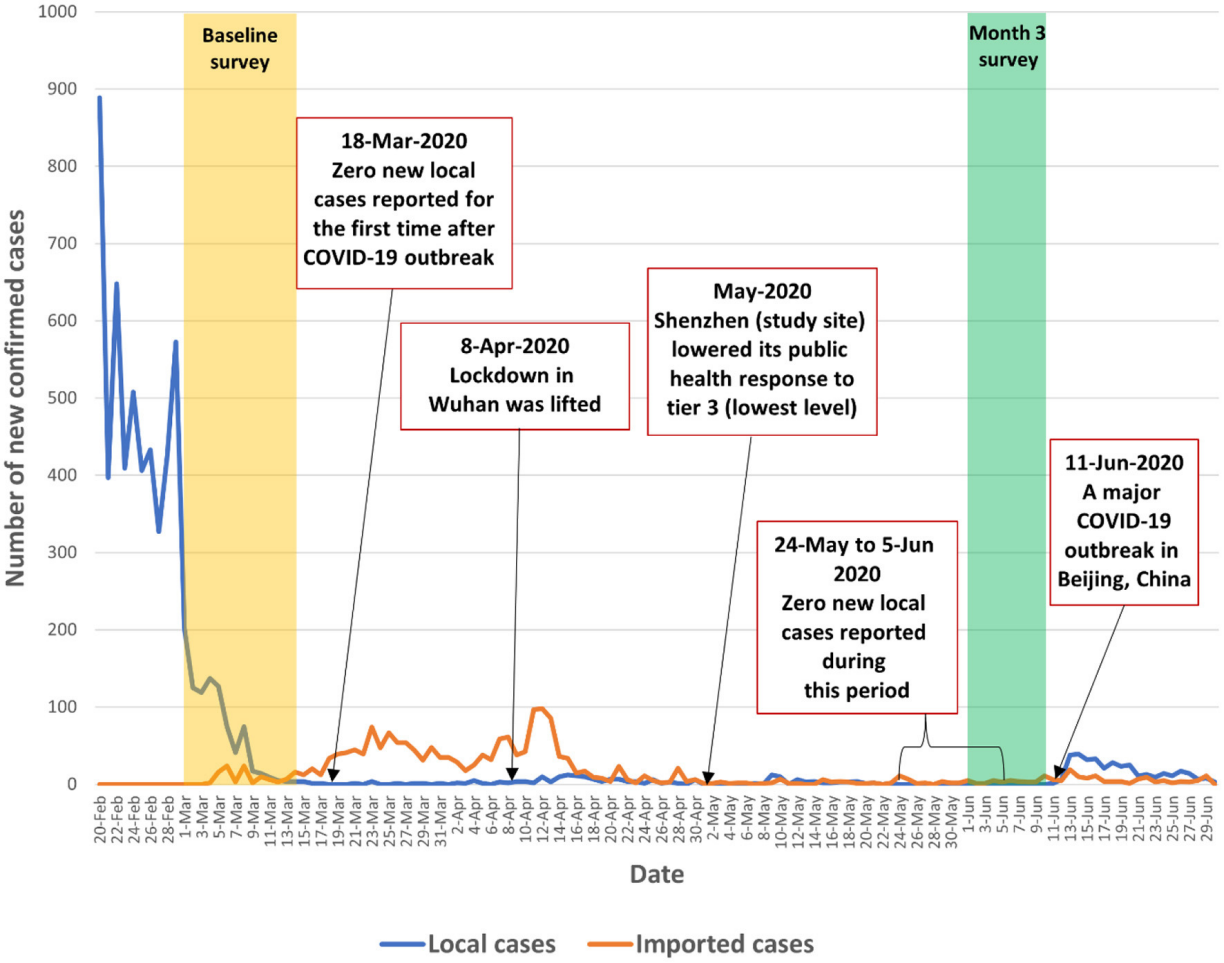
Number of newly confirmed COVID-19 cases during the study period in China.

**Figure 2:**
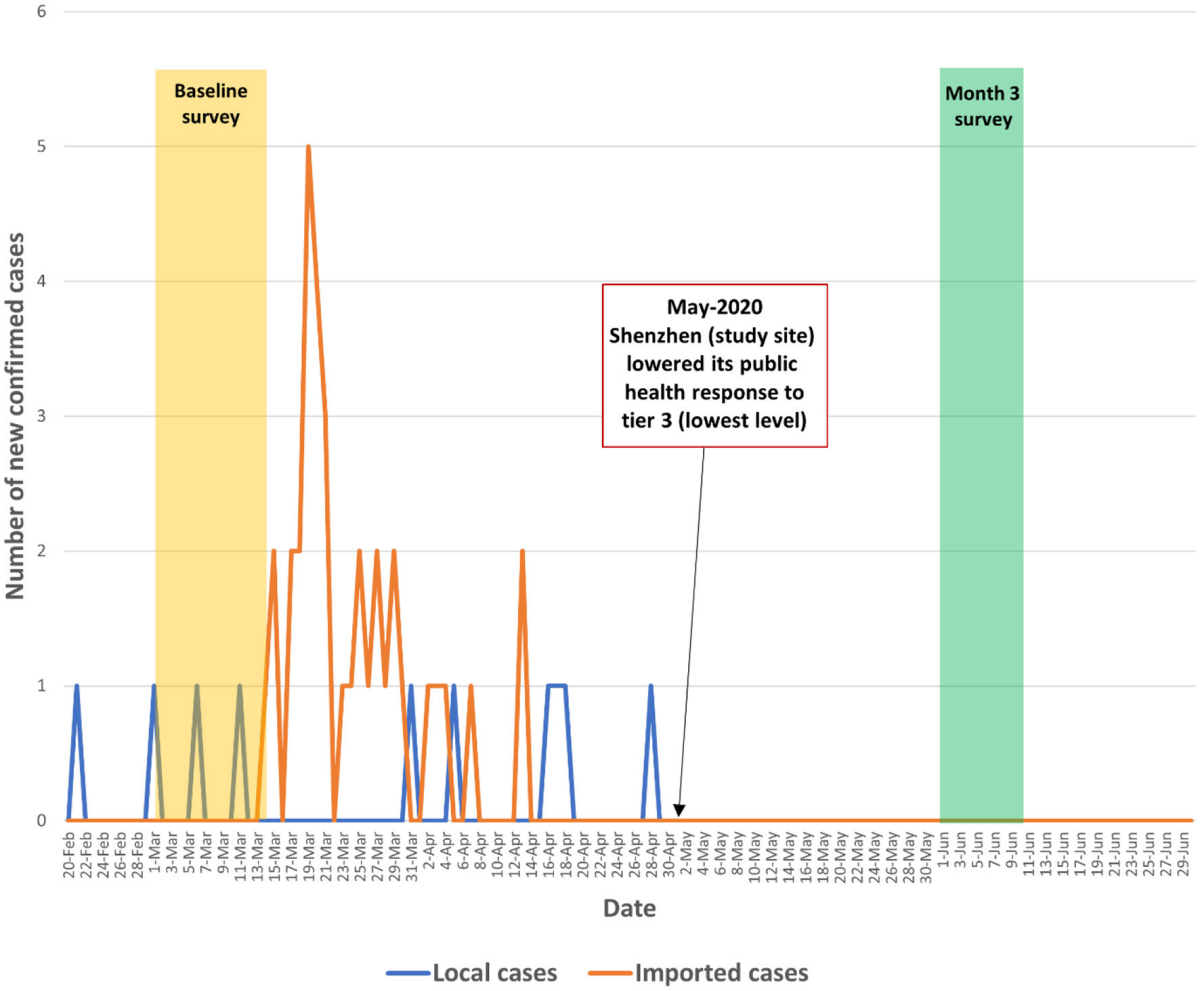
Number of newly confirmed COVID-19 cases during the study period in Shenzhen (the study site).

We conducted an observational prospective cohort study among factory workers in Shenzhen, China from March 1 to June 8, 2020. The baseline survey was conducted during March 1–14, 2020 when the number of daily confirmed cases began to decline from its peak in China (from 2,641 on February 14, 2020 to 202 on March 1, 2020). The number of daily confirmed cases continued to decline and the country recorded zero local new cases on March 18, 2020, which was the first time since the COVID-19 outbreak. The number of daily confirmed local cases remained low (0-12) between March 19 and June 12, 2020. The COVID-19 pandemic in mainland China was under initial control during the study period. The number of newly confirmed COVID-19 cases in China and in Shenzhen and the context of the present study were presented in Figures 1, 2 and Appendix 1.

### Participants and Sampling

Inclusion criteria for this cohort study were the following: (1) full-time employees aged ≥18 years who had resumed work, and (2) willing to leave contacts (mobile or social media account) to complete the follow-up survey. A stratified multi-stage cluster sampling design was used to recruit study participants. By March 1, 2020, 100 factories in Shenzhen had resumed work. In the first stage, the research team randomly selected 12 factories out of these 100 factories. These 12 factories manufactured electronic devices (*n* = 8), watches (*n* = 2), beverages (*n* = 1), and biotechnology products (*n* = 1). In the second stage, two workshops were randomly selected from each participating factory. In the final stage, all eligible workers in the selected workshops were invited to join the study. A typical workshop in these factories has 30–40 workers.

### Data Collection

In addition to national guidelines, the Shenzhen government requested that each factory set up WeChat groups covering all employees as part of preparations for work resumption (32, 33). Designated coordinators responsible for COVID-19 control in each factory facilitated the data collection. They posted the study information and the link to access the online self-administered baseline questionnaire in the WeChat groups, and invited all eligible workers in the selected workshops to participate. They also sent out four bi-weekly reminders in the WeChat groups. These designated coordinators did not participate in the actual survey. Before starting the baseline survey, participants read a statement indicating that participation was voluntary, refusal would have no effect on them, and data would only be used for research purposes. Online informed consent was obtained. The baseline survey took 10–15 min to complete. Participants were invited to complete another self-administered online questionnaire 3 months later. A link to access the online questionnaire was sent to participants *via* SMS. Five reminders were sent to participants at different time slots before the participant lost to follow-up. Upon completion of each survey, an e-coupon of 10RMB (1.3USD) was sent to participants as compensation for their time.

We developed the questionnaires using the Questionnaire Star, a commonly used online survey platform in China. The Questionnaire Star tool performed completeness checks before the questionnaires were submitted. Participants were able to review and change their responses through a “Back” button. Such approach could avoid incomplete questionnaires. Each individual WeChat account was allowed to access the online questionnaire once to avoid duplicate responses. Out of 950 workers in the selected workshops on March 1, 2020, 663 completed the baseline survey. The overall response rate was 69.8%. At Month 3, all 663 workers completed the follow-up survey. Ethics approval was obtained from the Seventh Affiliated Hospital, Sun Yat-sen University (reference: KY-2020-005-001).

### Measures

A panel consisting of two public health researchers, a health psychologist, two clinicians, a senior factory manager, and a factory worker was formed to develop the questionnaire used in the current study. The questionnaire was tested among 10 factory workers to assess clarity, readability, and length. All participants in the pilot testing agreed that the wording of the questions was appropriate and easy to understand and the length of the questionnaire was acceptable. The panel then finalized the questionnaire for the actual survey. These 10 workers did not participate in the actual survey.

Participants were asked to report on socio-demographics, such as age, gender, internal migrant status, highest education level, relationship status, monthly personal income, and status as frontline workers or management staff.

Compliance with six different personal preventive measures in the past month was measured at baseline and Month 3. Instruments used to measure facemask wearing in the workplace and other public settings, hand hygiene, physical distancing (avoiding social/meal gathering with people who do not live together and crowded places) were used in our previous studies targeting Chinese factory workers (1, 13). A single item was constructed for this study to measure the frequency of household disinfection (response categories: always, sometimes, seldom, never).

Depressive symptoms were measured using the validated Chinese version of Patient Health Questionnaire (PHQ-9) (34). Participants were asked to rate the frequency of experiencing each of nine depressive symptoms over the past 2 weeks (0 = never to 3 = nearly every day (alpha reliability = 0.91). We used a cut-off score of 7 to define probable depression. A previous study conducted among the general population in China recommended a cut-off score of 7 for the Chinese version of PHQ-9 for screening for probable depression (35). Global sleep quality over a 7-day recall period was measured using a single-item Sleep Quality Scale with a rating range from 0 to 10. A higher score indicated better sleep quality (36). This instrument has been demonstrated to be a reliable and valid measure without significantly increasing respondents' burden. The Chinese and English version of the baseline and follow-up questionnaire were in Appendix 2.

### Data Analysis

Descriptive data on all studied variables were presented. Normality tests were conducted for continuous variables (i.e., scores of the PHQ-9 scale and the Single-item Sleep Quality Scale). Changes in compliance with personal preventive measures, depressive symptoms, and sleep quality over the follow-up period were investigated using McNemar tests (for categorical variables) or paired sample *t*-test (for continuous variables).

We applied multilevel logistic/linear regression models in this study (level 1: factories; level 2: individual participants). Random intercept models were used to allow the intercept of the regression model to vary across factories, which could account for intra-correlated nested data. Multilevel logistic/linear regression models are commonly used in studies using similar cluster sampling methods (1, 37, 38).

Using compliance with six personal preventive measures measured at Month 3 as dependent variables, and baseline characteristics (sociodemographic, compliance with personal preventive measures at baseline, and scores of the PHQ-9 Scale and the Single-item Sleep Quality Scale at baseline) as independent variables, univariate multilevel logistic regression models were performed. Crude odds ratios (OR) and their 95% confidence interval (CI) were obtained. Multivariate multilevel logistic regression models were performed using all baseline characteristics as candidates. Adjusted OR (AOR) were obtained.

We log transformed the scores of the PHQ-9 Scale and the Single-item Sleep Quality Scale and used them as dependent variables. Associations between baseline characteristics (sociodemographic, compliance with personal preventive measures at baseline, and scores of the PHQ-9 scale and the Single-item Sleep Quality Scale at baseline) were analyzed using univariate multilevel linear regression models. Unadjusted unstandardized coefficients (B) were obtained. Using all baseline characteristics as candidates, adjusted B were obtained by using multivariate multilevel linear regression model.

In addition, we compared the score of the PHQ-9 Scale and the Single-item Sleep Quality Scale in four different subgroups of participants. These subgroups were those who complied with a personal measure at: (1) both baseline and Month 3, (2) only baseline, (3) only Month 3, and (4) neither baseline nor Month 3. SPSS version 23.0 for Windows (SPSS, Inc., Chicago, IL, the United States) was used for data analysis, with *P* < 0.05 considered statistically significant.

## Results

### Baseline Characteristics

About half of the participants were younger than 30 years old (51.3%), male (57.0%), married (58.8%), frontline workers (52.5%), and did not receive tertiary education (58.5%). Most of them were internal migrants (97.7%). At baseline, 98.0 and 97.1% of participants wore a facemask every time in the workplace and in other public spaces in the past month. Sanitizing hands every time after returning from public spaces or touching public installation (70.9%), avoiding social/meal gathering (76.6%), avoiding crowded places (69.8%) and always disinfect household (47.7%) were less common. The median and interquartile range (IQR) of the score of the PHQ-9 scale and the single-item Sleep Quality were 0 (IQR: 0, 3) and 8 (IQR: 6, 9), respectively (Table 1).

**Table 1:** Baseline characteristics of factory workers (*n* = 663).

	***n*** **(%)**
**Socio-demographics**	
Age group (years)	
18–25	125 (18.9)
26–30	215 (32.4)
31–40	272 (41.0)
>40	51 (7.7)
Gender	
Male	378 (57.0)
Female	285 (43.0)
Internal migrants	
Yes	648 (97.7)
No	15 (2.3)
Relationships status	
Currently single	203 (30.6)
Having a stable boyfriend/girlfriend	70 (10.6)
Married	390 (58.8)
Highest education level attained	
Junior high or below	208 (31.4)
Senior high or equivalent	180 (27.1)
College or university	234 (35.3)
Postgraduate	41 (6.2)
Monthly personal income (RMB)	
<3,000	38 (5.7)
3,000–4,999	249 (37.6)
5,000–9,999	261 (39.4)
≥10,000	115 (17.3)
Type of work	
Frontline workers	348 (52.5)
Management staff	315 (47.5)
**Compliance with personal preventive measures in the past month**	
Frequency of facemask wearing in workplace	650 (98.0)
Every time	
Often	11 (1.7)
Sometimes	1 (0.2)
Never	1 (0.2)
Frequency of facemask wearing in public places/transportation other than workplace	
Every time	644 (97.1)
Often	17 (2.6)
Sometimes	1 (0.2)
Never	1 (0.2)
Sanitizing hands (using soaps, liquid soaps or alcohol-based sanitizer) after returning from public spaces or touching public installation	
Every time	470 (70.9)
Often	113 (17.0)
Sometimes	57 (8.6)
Never	23 (3.5)
Avoided social/meal gathering with other people who do not live together	
No	155 (23.4)
Yes	508 (76.6)
Avoided crowded places	
No	200 (30.2)
Yes	463 (69.8)
Frequency of household disinfection	
Always	316 (47.7)
Sometimes	232 (35.0)
Seldom	86 (13.0)
Never	29 (4.4)
**Mental health status**	
PHQ-9 (Median/IQR)	0.0 (0.0, 3.0)
Sleep quality (Median/IQR)	8.0 (6.0, 9.0)

### Changes in Compliance With Personal Preventive Measures, Depressive Symptoms, and Sleep Quality Over the Follow-Up Period

Among 663 participants who completed both surveys, significant decline was observed in consistent facemask wearing in workplace (from 98.0 to 90.3%, *P* < 0.001), consistent facemask wearing in other public settings (from 97.1 to 94.4%, *P* = 0.02), sanitizing hands (from 70.9 to 48.0%, *P* < 0.001) and household disinfection (from 47.7 to 37.9%, *P* < 0.001). As compared to baseline data, more participants avoided crowded places at Month 3 (from 69.8 to 77.4%, *P* = 0.002). The change in avoiding social/meal gathering was not statistically significant (*P* = 0.18). Significant improvement in mental health was observed during the follow-up period, the prevalence of probable depression decreased from 14.9% at baseline to 1.5% at Month 3 (*P* < 0.001), and the proportion of participants reporting poor sleeping quality also dropped from 3.9% at baseline to 1.2% at Month 3 (*P* = 0.002) (Table 2).

**Table 2:** Changes in personal preventive measures and mental health status.

	**Baseline**	**Month 3**	**Percentage change (%)**	***P*** **-value**
	***n*** **(%)**	***n*** **(%)**		
**Compliance with personal preventive measures**				
Consistent facemask wearing in workplace				
No (never/sometimes/often)	13 (2.0)	64 (9.7)		
Yes (every time)	650 (98.0)	599 (90.3)	−7.9	<0.001
Consistent facemask wearing in public				
places/transportation other than workplace				
No (never/sometimes/often)	19 (2.9)	37 (5.6)		
Yes (every time)	644 (97.1)	626 (94.4)	−2.8	0.02
Types of facemask used				
Surgical masks	288 (43.4)	232 (35.0)	−19.4	0.001
Non-surgical grade respirators	454 (68.5)	452 (68.2)	−0.4	0.95
N-95 masks	187 (28.2)	58 (8.7)	−69.1	<0.001
Cloth masks	33 (5.0)	8 (1.2)	−76.0	<0.001
Re-used facemask				
No	512 (77.2)	607 (91.6)		
Yes	151 (22.8)	56 (8.4)	−63.2	<0.001
Sanitizing hands (using soaps, liquid soaps or alcohol-based sanitizer) after returning from public spaces or touching public installation				
Never/sometimes/often	193 (29.1)	345 (52.0)		
Every time	470 (70.9)	318 (48.0)	−32.3	<0.001
Avoided social/meal gathering with other people who do not live together				
No	155 (23.4)	134 (20.2)		
Yes	508 (76.6)	529 (79.8)	4.2	0.18
Avoided crowded places				
No	200 (30.2)	150 (22.6)		
Yes	463 (69.8)	513 (77.4)	10.9	0.002
Household disinfection				
Never/seldom/sometimes	347 (52.3)	412 (62.1)		
Always	316 (47.7)	251 (37.9)	−20.5	<0.001
**Mental health status**				
Score of the PHQ-9 Scale (median/IQR)	0.0 (0.0, 0.30)	0.0 (0.0, 0.0)	N.A.	<0.001
Probable depression (PHQ-9 score ≥7)				
No	564 (85.1)	653 (98.5)		
Yes	99 (14.9)	10 (1.5)	−90.0	<0.001
Score of the single-item Sleep Quality Scale (median/IQR)	8 (6, 9)	8 (8, 10)	N.A.	<0.001
Poor sleeping quality (sleep quality score ≤3)				
No	637 (96.1)	655 (98.8)		
Yes	26 (3.9)	8 (1.2)	−69.3	0.002

*N.A., not applicable*.

### Baseline Factors Predicting Compliance With Personal Preventive Measures at Month 3

In multivariate multilevel logistic regression models, those who had better sleep quality at baseline were more likely to wear facemask consistently in public spaces other than workplace at Month 3 (AOR: 1.17, 95%CI: 1.01, 1.36). Being female (AOR: 1.50, 95%CI: 1.03, 2.20) and always sanitizing hands at baseline (AOR: 2.17, 95%CI: 1.48, 3.19) were associated with higher frequency of sanitizing hands at Month 3, while higher education level (senior high or equivalent: AOR: 0.56, 95%CI: 0.35, 0.88; reference group: junior high or below) and depressive symptoms at baseline (AOR: 0.94, 95% CI: 0.89, 0.99) were negatively associated with this dependent variable. Moreover, older participants were less likely to avoid social/meal gathering at Month 3 (>40 years: AOR: 0.38, 95% CI: 0.15, 0.96; reference group: 18–25 years). Furthermore, always sanitizing hands (AOR: 2.06, 95% CI: 1.38, 3.08) and disinfecting household (AOR: 2.82, 95% CI: 1.00, 8.82) at baseline were positively associated with household disinfection at Month 3, while education level (college or university: AOR: 0.40, 95% CI: 0.22, 0.70; reference group: junior high or below) was negatively associated with this dependent variable (Table 3).

**Table 3:** Baseline factors predicting personal preventive behaviors at Month 3 (results of multivariate multilevel logistic regression models; level 1: factories, level 2: individual participants).

	**Consistent facemask wearing in workplace**	**Consistent facemask wearing in other public places**	**Sanitizing hands every time**	**Avoided social/meal gathering**	**Avoided crowded places**	**Always disinfect household**
	**AOR** **(95% CI)**	**AOR** **(95% CI)**	**AOR** **(95% CI)**	**AOR** **(95% CI)**	**AOR** **(95% CI)**	**AOR** **(95% CI)**
**Socio-demographics**						
Age group (years)						
18–25	Ref	Ref	Ref	Ref	Ref	Ref
26–30	0.95 (0.44, 2.05)	0.89 (0.36, 2.19)	0.76 (0.45, 1.29)	0.66 (0.34, 1.26)	1.13 (0.62, 2.07)	0.98 (0.58, 1.67)
31–40	0.96 (0.40, 2.29)	0.95 (0.36, 2.50)	1.06 (0.60, 1.87)	0.83 (0.41, 1.68)	1.15 (0.60, 2.23)	0.85 (0.48, 1.50)
>40	0.75 (0.22, 2.57)	0.99 (0.25, 4.01)	1.60 (0.70, 3.66)	0.38 (0.15, 0.96)^a^	0.75 (0.30, 1.86)	0.70 (0.31, 1.59)
Gender						
Male	Ref	Ref	Ref	Ref	Ref	Ref
Female	1.31 (0.72, 2.39)	1.19 (0.60, 2.33)	1.50 (1.03, 2.20)^a^	1.19 (0.75, 1.89)	1.04 (0.67, 1.63)	1.00 (0.68, 1.48)
Internal migrants						
Yes	Ref	Ref	Ref	Ref	Ref	Ref
No	0.46 (0.13, 1.67)	1.15 (0.16, 8.47)	0.82 (0.26, 2.61)	0.53 (0.16, 1.70)	0.66 (0.19, 2.33)	0.56 (0.15, 2.14)
Relationships status						
Currently single	Ref	Ref	Ref	Ref	Ref	Ref
Having a stable boyfriend/girlfriend	0.52 (0.24, 1.17)	1.15 (0.39, 3.44)	0.65 (0.35, 1.21)	1.49 (0.66, 3.35)	0.81 (0.41, 1.61)	0.77 (0.41, 1.46)
Married	1.18 (0.58, 2.40)	0.99 (0.45, 2.17)	0.80 (0.51, 1.26)	0.84 (0.49, 1.43)	0.68 (0.40, 1.16)	0.93 (0.58, 1.47)
Highest education level attained						
Junior high or below	Ref	Ref	Ref	Ref	Ref	Ref
Senior high or equivalent	1.10 (0.50, 2.40)	2.22 (1.00, 5.01)^a^	0.56 (0.35, 0.88)^a^	1.24 (0.70, 2.17)	1.03 (0.61, 1.76)	0.93 (0.59, 1.46)
College or university	0.72 (0.30, 1.70)	1.92 (0.74, 4.97)	0.77 (0.34, 1.74)	0.89 (0.46, 1.74)	1.04 (0.53, 2.04)	0.40 (0.22, 0.70)^b^
Postgraduate	2.77 (0.51, 15.06)	4.81 (0.70, 33.17)	0.71 (0.26, 1.94)	0.99 (0.31, 3.13)	0.63 (0.21, 1.89)	0.38 (0.13, 1.05)
Monthly personal income (RMB)						
<3,000	Ref	Ref	Ref	Ref	Ref	Ref
3,000–4,999	2.58 (0.90, 7.39)	1.21 (0.38, 3.83)	0.66 (0.31, 1.41)	1.11 (0.46, 2.69)	0.65 (0.26, 1.62)	1.01 (0.49, 2.09)
5,000–9,999	2.05 (0.66, 6.38)	2.07 (0.55, 7.82)	0.77 (0.34, 1.74)	1.02 (0.39, 2.68)	0.71 (0.26, 1.93)	0.94 (0.43, 2.07)
≥10,000	1.80 (0.43. 7.51)	0.97 (0.19, 5.00)	0.71 (0.26, 1.94)	0.87 (0.26, 2.87)	0.45 (0.13, 1.52)	0.92 (0.34, 2.54)
Type of work						
Frontline workers	Ref	Ref	Ref	Ref	Ref	Ref
Management staff	0.91 (0.45, 1.83)	0.86 (0.38, 1.93)	1.33 (0.84, 2.11)	1.52 (0.87, 2.66)	1.65 (0.96, 2.84)	1.05 (0.66, 1.67)
**Compliance with personal preventive measures at baseline**						
Consistent facemask wearing in workplace						
No	Ref	Ref	Ref	Ref	Ref	Ref
Yes	2.78 (0.74, 10.38)	1.88 (0.38, 9.69)	3.98 (0.80, 19.73)	0.48 (0.10, 2.34)	0.75 (0.19, 3.04)	1.27 (0.31, 5.20)
Consistent facemask wearing in other public places						
No	Ref	Ref	Ref	Ref	Ref	Ref
Yes	0.76 (0.11, 5.35)	0.62 (0.09, 4.38)	0.36 (0.12, 1.08)	1.18 (0.38, 3.68)	1.17 (0.40, 3.45)	0.41 (0.15, 1.14)
Sanitizing hands after returning from public spaces or touching public installation						
Never/sometimes/often	Ref	Ref	Ref	Ref	Ref	Ref
Every time	0.93 (0.52, 1.65)	0.79 (0.41, 1.53)	2.17 (1.48, 3.19)^c^	0.95 (0.61, 1.49)	1.34 (0.88, 2.05)	2.06 (1.38, 3.08)^c^
Avoided social/meal gathering with other people who do not live together						
No	Ref	Ref	Ref	Ref	Ref	Ref
Yes	0.65 (0.27, 1.57)	1.37 (0.51, 3.70)	1.21 (0.68, 2.17)	1.03 (0.53, 2.00)	1.36 (0.71, 2.60)	1.08 (0.61, 1.91)
Avoided crowded places						
No	Ref	Ref	Ref	Ref	Ref	Ref
Yes	1.32 (0.59, 2.97)	0.63 (0.24, 1.66)	0.60 (0.35, 1.04)	1.27 (0.68, 2.39)	0.98 (0.53, 1.84)	0.87 (0.51, 1.49)
Household disinfection						
Never/seldom/sometimes	Ref	Ref	Ref	Ref	Ref	Ref
Always	1.20 (0.33, 4.31)	0.99 (0.23, 4.27)	1.81 (0.22, 1.37)	2.34 (0.99, 5.59)	1.69 (0.72, 3.94)	2.82 (1.00, 8.82)^a^
**Mental health status**						
Score of the PHQ-9 Scale	1.01 (0.94, 1.08)	1.01 (0.93, 1.09)	0.94 (0.89, 0.99)^a^	1.00 (0.94, 1.06)	0.98 (0.93, 1.04)	0.99 (0.94, 1.04)
Score of the single-item Sleep Quality Scale	1.11 (0.96, 1.27)	1.17 (1.01, 1.36)^a^	0.94 (0.86, 1.04)	1.08 (0.96, 1.20)	1.05 (0.94, 1.16)	0.98 (0.89, 1.08)

*AOR, adjusted odds ratios, odds ratios obtained from two-level logistic regression models (level 1: factories, level 2: individual participants) using all variables listed in Table 1 as candidates*.

a *P < 0.05*,

b *P < 0.01*,

c *P < 0.001*.

### Baseline Factors Predicting Depressive Symptoms and Sleep Quality at Month 3

Multivariate multilevel linear regression models showed that those who had higher depressive symptoms at baseline were more likely to have depressive symptoms at Month 3 (adjusted B: 0.01, 95% CI: 0.00, 0.03). Having higher education level (adjusted B: −0.01, 95% CI: −0.02, −0.00) and depressive symptoms (adjusted B: −0.004, 95% CI: −0.01, −0.002) at baseline were associated with poorer sleep quality at Month 3, while being management staff (adjusted B: 0.03, 95% CI: 0.01, 0.04) and having better sleep quality (adjusted B: 0.01, 95% CI: 0.002, 0.01) at baseline were associated with better sleep quality during the follow-up period (Table 4).

**Table 4:** Baseline factors predicting depressive symptoms and sleeping quality at Month 3 (results of univariate and multivariate multilevel linear regression models; level 1: factories; level 2: individual participants).

	**Log transformation of the score of the PHQ-9 scale with a base of 10**		**Log transformation of the score of the Single-item Sleep Quality Scale with a base of 10**	
	**B** **(95% CI)**	**Adjusted B** **(95% CI)**	**B** **(95% CI)**	**Adjusted B** **(95% CI)**
**Socio-demographics**				
Age group	−0.07 (−0.12, −0.01)^a^	−0.02 (−0.10, 0.05)	0.01 (−0.001, 0.02)	−0.003 (−0.01, 0.01)
Gender	−0.5 (−0.15, 0.05)	−0.05 (−0.16, 0.06)	0.01 (−0.003, 0.03)	0.01 (−0.001, 0.03)
Internal migrants	0.02 (−0.26, 0.30)	−0.02 (−0.32, 0.28)	−0.01 (−0.06, 0.04)	0.01 (−0.04, 0.06)
Relationships status	−0.06 (−0.11, 0.002)	−0.02 (−0.09, 0.05)	0.10 (0.002, 0.02)^a^	0.001 (−0.01, 0.01)
Highest education level attained	0.01 (−0.04, 0.06)	0.04 (−0.04, 0.12)	−0.01 (−0.02, −0.001)^a^	−0.01 (−0.02, −0.00)^a^
Monthly personal income (RMB)	−0.03 (−0.07, 0.01)	−0.04 (−0.10. 0.03)	0.003 (−0.004, 0.01)	0.01 (−0.004, 0.02)
Type of work	−0.04 (−0.14, 0.06)	−0.03 (−0.16, 0.10)	0.01 (−0.004, 0.03)	0.03 (0.01, 0.04)^a^
**Compliance with personal preventive measures in the past month**				
Consistent facemask wearing in workplace	−0.12 (−0.43, 0.19)	−0.02 (−0.39, 0.35)	0.01 (−0.04, 0.06)	0.01 (−0.04, 0.06)
Consistent facemask wearing in other public places	−0.04 (−0.35, 0.27)	−0.04 (−0.38, 0.31)	−0.02 (−0.06, 0.02)	−0.02 (−0.06, 0.02)
Sanitizing hands after returning from public spaces or touching public installation	−0.07 (−0.18, 0.03)	−0.04 (−0.15, 0.07)	0.01 (−0.01, 0.02)	−0.01 (−0.02, 0.01)
Avoided social/meal gathering with other people who do not live together	−0.07 (−0.19, 0.06)	−0.04 (−0.19, 0.09)	−0.02 (−0.03, −0.001)^a^	−0.02 (−0.05, 0.002)
Avoided crowded places	−0.05 (−0.16, 0.05)	−0.05 (−0.19, 0.09)	−0.01 (−0.03, 0.01)	0.01 (−0.02, 0.03)
Household disinfection	0.15 (−0.14, 0.05)	0.003 (−0.10,0.11)	0.01 (−0.01, 0.02)	0.00 (−0.01, 0.02)
**Mental health status**				
PHQ-9	0.02 (0.01, 0.03)^c^	0.01 (0.00, 0.03)^a^	−0.01 (−0.007, −0.004)^c^	−0.004 (−0.01, −0.002)^c^
Sleep quality	−0.03 (−0.05, −0.003)^c^	−0.01 (−0.04, 0.02)	0.01 (0.007, 0.013)^c^	0.01 (0.002, 0.01)^b^

a *P < 0.05*,

b *P < 0.01*,

c *P < 0.001*.

*B, unstandardized coefficients obtained from multilevel linear regression models (level 1: factories, level 2: individual participants)*.

*Score of PHQ-9 and the single-item Sleep Quality Scale were log transformed using the base of 10*.

*Adjusted B, adjusted unstandardized coefficients obtained by multilevel multivariate linear regression model (level 1: factories, level 2: individual participants) using all variables listed in Table 1 as candidates*.

### Comparing Depressive Symptoms and Sleep Quality Between Subgroups With Different Compliance With Personal Preventive Measures at Baseline and Month 3

The results showed that as compared to factory workers who complied to facemask wearing at workplace at both baseline and Month 3, those who only did it at baseline but not at Month 3 had poorer sleep quality at Month 3 (B: −0.05, 95% CI: −0.07, −0.02). There was no difference in the scores of the PHQ-9 and the single-item Sleep Quality Scale between other subgroups (Appendix 3).

## Discussion

We reported a longitudinal study to track change in compliance with personal preventive measures and mental health from the early phase of the outbreak to the initial control of the COVID-19 pandemic in China, providing potentially important new information for intervention development and policy planning. The results confirmed our concerns that people's compliance with some personal preventive measures declined when the pandemic is under initial control. Significant decline in compliance with consistent facemask wearing was observed, especially in workplace. In workplace where physical distancing cannot be guaranteed, consistent facemask wearing is especially important for COVID-19 control. In the early phase of the outbreak, factories in China were implementing very strict measures, including providing free facemask for employees and requiring consistent facemask wearing in workplace (32, 33), which might explain the very high prevalence of consistent facemask wearing at the baseline. It is possible that these strict control measures are lifted when the pandemic is under initial control. Since the risk of an outbreak in workplace always exists, factories should maintain effective measures to ensure high compliance with consistent facemask wearing during the pandemic. Although the guideline on facemask wearing established by the Chinese Centre for Disease Control and Prevention no longer required consistent facemask wearing in public spaces where physical distancing can be guaranteed (39), 94.4% of factory workers reported consistent facemask wearing in public spaces other than workplace. As compared to baseline data, fewer participants used surgical masks and N-95 masks. It is possible that more factory workers followed the aforementioned guideline which recommended non-surgical grade respirators in settings where the risk of COVID-19 transmission was relatively low (e.g., workplace) (39). Facemask was in limited supply in China in the early phase of the COVID-19 outbreak (40, 41). The supply issue was quickly addressed, as China had largely increased its facemask wearing capacity (42, 43). It is hence expected that fewer participants used cloth mask or re-used facemask at Month 3.

Despite the WHO recommendation on hand hygiene (8), only 70% of participants always sanitized their hands at baseline, such proportion dropped substantially to <50% at Month 3. The importance of hand hygiene might be less emphasized than consistent facemask wearing in China during the outbreak. Moreover, there might be a lack of appropriate places for workers to sanitize their hands, especially in workplace. Similarly, regular household disinfection was less common and was declining over time. The majority of the factory workers in Shenzhen are internal migrants who lived in dormitories provided by the employers. Factories should pay more attention to the hygiene of these dormitories, as COVID-19 outbreak in tightly-packed dormitories of foreign workers occurred in Singapore (44). In contrast to the aforementioned personal preventive behaviors, physical distancing behaviors remained stable, or even slightly increased over time. Such trends might reflect the nationwide health education and health promotion of physical distancing (45).

Our study observed significant improvement in both depressive symptoms and sleep quality among the participants. Although the COVID-19 outbreak triggered mental health problems among participants at the beginning of work resumption, prevalence of moderate-to-severe depression and poor sleep quality dropped substantially at Month 3 when the pandemic in China is under initial control. The prevalence of moderate-to-severe depressive symptoms was even lower than the population-level observed before the pandemic (2.4%) (46). One possible explanation was that most of the factory workers were younger and relatively healthier than the general population (47). The results also indicated that mental health problems triggered by the COVID-19 among factory workers might be one-off, and would improve without any intervention after the pandemic received initial control.

Our first hypothesis that poorer mental health status at baseline was associated with poorer compliance with personal preventive measures were partially supported by the results. Higher depressive symptoms at the baseline was associated with poor compliance with hand hygiene, while better sleep quality was associated with better compliance with facemask wearing in public spaces other than workplace. Such findings were similar to those obtained in cross-sectional studies (1, 30, 31). As suggested by a previous study, characteristics associated with depression, such as lower levels of energy, a decreased focus, and greater hopelessness might result in disengagement in hand hygiene during COVID-19 (29). However, our second hypothesis was not supported by our data, as the associations between compliance with personal preventive measures at baseline and mental health outcomes at Month 3 were all statistically non-significant. However, we found that a drop in compliance with facemask wearing in workplace was associated with poorer sleep quality at Month 3.

Our study also provided empirical insights to inform intervention development to strengthen compliance with personal preventive measures during the pandemic, and suggested the need to tailor interventions to specific groups. Male factory workers were less likely to sanitize hands regularly, promotion efforts should take gender difference into account. Health promotion targeting workers with higher education should focus on hand and household hygiene. Participants who always sanitized hands and disinfect household at baseline were more likely to do so at Month 3. Participants who made handwashing and household disinfection a habit might be more likely to maintain these behaviors during the COVID-19 pandemic. Creating a supportive environment is important to converting a behavior into a habit. Factories should consider strategically placing hand sanitizer in high traffic locations throughout the workplace, and distributing household bleach and tools regularly to workers' dormitories. Baseline depressive symptoms and poor sleep quality was associated with the presence of these two mental health problems during the follow-up period. Such finding was consistent with those of longitudinal studies conducted in the UK (17). Studies suggested that personality traits such as neuroticism and negative thinking pattern are strong factors of mental health problems during the COVID-19 (48). Similar to findings of previous cross-sectional studies, baseline depressive symptoms was a risk factor of sleep quality (22). In addition, higher education level was also associated with poorer sleep quality at Month 3. Previous studies showed that higher education can increase negative emotions and sleep problems during public health emergencies, probably due to high self-awareness of their health (22). In contrast, being management staff was associated with better sleep quality. Most management staff are white-collar workers who generally have better sleep quality than blue-collar workers (22). An alternative explanation was that management staff had higher and more stable income as compared to frontline workers. Studies showed that concerns about pay cut or layoffs due to COVID-19 caused stress among workers (22).

However, this study had some limitations. First, we only included factory workers in one Chinese city. Generalization should be made cautiously to individuals working in other types of enterprises or to other places in China. Second, non-response may introduce selection bias. Our response rate was relatively high (70.0%) as compared to other online surveys of similar topics (19, 20). We were not able to collect information on workers who refused to participate in the study. Therefore, we were not able to wright our findings to estimate the situation of all factory employees. Our sample might not be representative of factory workers in Shenzhen. Third, data were self-reported and verification was not feasible. Recall bias might exist. Participants might also over-report their compliance with personal preventive measures due to social desirability. Fourth, the findings would be different if the follow-up period was longer, as there was a major COVID-19 outbreak shortly after the completion of the Month 3 follow-up survey. The relatively short follow-up period was another limitation of this study. Fifth, we did not employ measures to avoid repeated choices for all options in the questionnaires. However, we did not observe such situation in our study. Moreover, we did not collect information on whether certain types of work should wear facemask due to work needs instead of COVID-19 prevention. Furthermore, we did not include important mental health outcomes such as anxiety due to the limited space of the questionnaires. Anxiety was considered as a more sensitive mental health indicator during the COVID-19 pandemic.

## Conclusions

In sum, consistent facemask wearing, hand hygiene, and household disinfection declined significantly during the follow-up period. Health promotion is needed to strengthen these preventive measures, and more attention should be given to male workers and those with high education level. Mental health problems were uncommon and likely to be one-off among Chinese factory workers. Both depressive symptoms and sleep quality improved significantly after the COVID-19 pandemic received initial control in mainland China.

## Data Availability Statement

The raw data supporting the conclusions of this article will be made available by the authors, without undue reservation.

## Ethics Statement

The studies involving human participants were reviewed and approved by Seventh Affiliated Hospital, Sun Yat-sen University (reference: KY-2020-005-001). The patients/participants provided their online informed consent to participate in this study.

## Author Contributions

JY, BC, YP, YH, and ZW conceived the idea. JY, BC, and ZW designed the methodology and conducted formal analysis. CZ, PC, MX, YF, YC, DH, LL, XX, and GZ participated in the investigation. CZ, PC, MX, YF, YC, DH, LL, XX, and GZ carried out data curation. YF and ZW drafted the original manuscript. JY, BC, YP, YH, and ZW reviewed and edited the manuscript. All authors have read and agreed to submission version of the manuscript. All authors contributed to the article and approved the submitted version.

## Funding

This study was funded by the Startup Fund of 100 Top Talents Program, Sun Yat-sen University (grant Number 392012) and the National Key Research and Development Program (grant number 2018YFA0902801).

## Conflict of Interest

The authors declare that the research was conducted in the absence of any commercial or financial relationships that could be construed as a potential conflict of interest.

## Publisher's Note

All claims expressed in this article are solely those of the authors and do not necessarily represent those of their affiliated organizations, or those of the publisher, the editors and the reviewers. Any product that may be evaluated in this article, or claim that may be made by its manufacturer, is not guaranteed or endorsed by the publisher.
