# Supplementary material for: Changes in Compliance With Personal Preventive Measures and Mental Health Status Among Chinese Factory Workers During the COVID-19 Pandemic: An Observational Prospective Cohort Study
Source: Front Public Health. 2022 Mar 10;10:831456. doi: 10.3389/fpubh.2022.831456 (PMC8960195; doi:10.3389/fpubh.2022.831456)
Supplement: Supplementary file 1 [file Table_1.DOCX]

Appendix 1 Number of newly confirmed COVID-19 cases from 20-Feb to 30-June-2020 in Shenzhen and China.

| Date  (2020) | Shenzhen | | | China | | |
| --- | --- | --- | --- | --- | --- | --- |
|  | Local cases | Imported cases | Total | Local cases | Imported cases | Total |
| 20-Feb | 0 | 0 | 0 | 889 | 0 | 889 |
| 21-Feb | 1 | 0 | 1 | 397 | 0 | 397 |
| 22-Feb | 0 | 0 | 0 | 648 | 0 | 648 |
| 23-Feb | 0 | 0 | 0 | 409 | 0 | 409 |
| 24-Feb | 0 | 0 | 0 | 508 | 0 | 508 |
| 25-Feb | 0 | 0 | 0 | 406 | 0 | 406 |
| 26-Feb | 0 | 0 | 0 | 433 | 0 | 433 |
| 27-Feb | 0 | 0 | 0 | 327 | 0 | 327 |
| 28-Feb | 0 | 0 | 0 | 427 | 0 | 427 |
| 29-Feb | 0 | 0 | 0 | 573 | 0 | 573 |
| 1-Mar | 1 | 0 | 1 | 202 | 0 | 202 |
| 2-Mar | 0 | 0 | 0 | 125 | 0 | 125 |
| 3-Mar | 0 | 0 | 0 | 119 | 0 | 119 |
| 4-Mar | 0 | 0 | 0 | 137 | 2 | 139 |
| 5-Mar | 0 | 0 | 0 | 127 | 16 | 143 |
| 6-Mar | 1 | 0 | 1 | 75 | 24 | 99 |
| 7-Mar | 0 | 0 | 0 | 41 | 3 | 44 |
| 8-Mar | 0 | 0 | 0 | 75 | 24 | 99 |
| 9-Mar | 0 | 0 | 0 | 17 | 2 | 19 |
| 10-Mar | 0 | 0 | 0 | 14 | 10 | 24 |
| 11-Mar | 1 | 0 | 1 | 9 | 6 | 15 |
| 12-Mar | 0 | 0 | 0 | 5 | 3 | 8 |
| 13-Mar | 0 | 0 | 0 | 4 | 7 | 11 |
| 14-Mar | 0 | 1 | 1 | 4 | 16 | 20 |
| 15-Mar | 0 | 2 | 2 | 4 | 12 | 16 |
| 16-Mar | 0 | 0 | 0 | 1 | 20 | 21 |
| 17-Mar | 0 | 2 | 2 | 1 | 12 | 13 |
| 18-Mar | 0 | 2 | 2 | 0 | 34 | 34 |
| 19-Mar | 0 | 5 | 5 | 0 | 39 | 39 |
| 20-Mar | 0 | 4 | 4 | 0 | 41 | 41 |
| 21-Mar | 0 | 3 | 3 | 1 | 45 | 46 |
| 22-Mar | 0 | 0 | 0 | 0 | 39 | 39 |
| 23-Mar | 0 | 1 | 1 | 4 | 74 | 78 |
| 24-Mar | 0 | 1 | 1 | 0 | 47 | 47 |
| 25-Mar | 0 | 2 | 2 | 0 | 67 | 67 |
| 26-Mar | 0 | 1 | 1 | 1 | 54 | 55 |
| 27-Mar | 0 | 2 | 2 | 0 | 54 | 54 |
| 28-Mar | 0 | 1 | 1 | 1 | 44 | 45 |
| 29-Mar | 0 | 2 | 2 | 1 | 31 | 32 |
| 30-Mar | 0 | 1 | 1 | 0 | 48 | 48 |
| 31-Mar | 1 | 0 | 1 | 1 | 35 | 36 |
| 1-Apr | 0 | 0 | 0 | 0 | 35 | 35 |
| 2-Apr | 0 | 1 | 1 | 2 | 29 | 31 |
| 3-Apr | 0 | 1 | 1 | 1 | 18 | 19 |
| 4-Apr | 0 | 1 | 1 | 5 | 25 | 30 |
| 5-Apr | 1 | 0 | 1 | 1 | 38 | 39 |
| 6-Apr | 0 | 0 | 0 | 0 | 32 | 32 |
| 7-Apr | 0 | 1 | 1 | 3 | 59 | 62 |
| 8-Apr | 0 | 0 | 0 | 2 | 61 | 63 |
| 9-Apr | 0 | 0 | 0 | 4 | 38 | 42 |
| 10-Apr | 0 | 0 | 0 | 4 | 42 | 46 |
| 11-Apr | 0 | 0 | 0 | 2 | 97 | 99 |
| 12-Apr | 0 | 0 | 0 | 10 | 98 | 108 |
| 13-Apr | 0 | 2 | 2 | 3 | 86 | 89 |
| 14-Apr | 0 | 0 | 0 | 10 | 36 | 46 |
| 15-Apr | 0 | 0 | 0 | 12 | 34 | 46 |
| 16-Apr | 1 | 0 | 1 | 11 | 15 | 26 |
| 17-Apr | 1 | 0 | 1 | 10 | 17 | 27 |
| 18-Apr | 1 | 0 | 1 | 7 | 9 | 16 |
| 19-Apr | 0 | 0 | 0 | 4 | 8 | 12 |
| 20-Apr | 0 | 0 | 0 | 7 | 4 | 11 |
| 21-Apr | 0 | 0 | 0 | 7 | 23 | 30 |
| 22-Apr | 0 | 0 | 0 | 4 | 6 | 10 |
| 23-Apr | 0 | 0 | 0 | 4 | 2 | 6 |
| 24-Apr | 0 | 0 | 0 | 1 | 11 | 12 |
| 25-Apr | 0 | 0 | 0 | 6 | 5 | 11 |
| 26-Apr | 0 | 0 | 0 | 1 | 2 | 3 |
| 27-Apr | 0 | 0 | 0 | 3 | 3 | 6 |
| 28-Apr | 1 | 0 | 1 | 1 | 21 | 22 |
| 29-Apr | 0 | 0 | 0 | 0 | 4 | 4 |
| 30-Apr | 0 | 0 | 0 | 6 | 6 | 12 |
| 1-May | 0 | 0 | 0 | 0 | 1 | 1 |
| 2-May | 0 | 0 | 0 | 1 | 1 | 2 |
| 3-May | 0 | 0 | 0 | 0 | 3 | 3 |
| 4-May | 0 | 0 | 0 | 0 | 1 | 1 |
| 5-May | 0 | 0 | 0 | 0 | 2 | 2 |
| 6-May | 0 | 0 | 0 | 0 | 2 | 2 |
| 7-May | 0 | 0 | 0 | 1 | 0 | 1 |
| 8-May | 0 | 0 | 0 | 0 | 1 | 1 |
| 9-May | 0 | 0 | 0 | 12 | 2 | 14 |
| 10-May | 0 | 0 | 0 | 10 | 7 | 17 |
| 11-May | 0 | 0 | 0 | 0 | 1 | 1 |
| 12-May | 0 | 0 | 0 | 6 | 1 | 7 |
| 13-May | 0 | 0 | 0 | 3 | 0 | 3 |
| 14-May | 0 | 0 | 0 | 4 | 0 | 4 |
| 15-May | 0 | 0 | 0 | 2 | 6 | 8 |
| 16-May | 0 | 0 | 0 | 2 | 3 | 5 |
| 17-May | 0 | 0 | 0 | 3 | 4 | 7 |
| 18-May | 0 | 0 | 0 | 3 | 3 | 6 |
| 19-May | 0 | 0 | 0 | 4 | 1 | 5 |
| 20-May | 0 | 0 | 0 | 1 | 1 | 2 |
| 21-May | 0 | 0 | 0 | 2 | 2 | 4 |
| 22-May | 0 | 0 | 0 | 0 | 0 | 0 |
| 23-May | 0 | 0 | 0 | 1 | 2 | 3 |
| 24-May | 0 | 0 | 0 | 0 | 11 | 11 |
| 25-May | 0 | 0 | 0 | 0 | 7 | 7 |
| 26-May | 0 | 0 | 0 | 0 | 1 | 1 |
| 27-May | 0 | 0 | 0 | 0 | 2 | 2 |
| 28-May | 0 | 0 | 0 | 0 | 0 | 0 |
| 29-May | 0 | 0 | 0 | 0 | 4 | 4 |
| 30-May | 0 | 0 | 0 | 0 | 2 | 2 |
| 31-May | 0 | 0 | 0 | 0 | 2 | 2 |
| 1-Jun | 0 | 0 | 0 | 0 | 5 | 5 |
| 2-Jun | 0 | 0 | 0 | 0 | 1 | 1 |
| 3-Jun | 0 | 0 | 0 | 0 | 1 | 1 |
| 4-Jun | 0 | 0 | 0 | 0 | 5 | 5 |
| 5-Jun | 0 | 0 | 0 | 0 | 3 | 3 |
| 6-Jun | 0 | 0 | 0 | 1 | 5 | 6 |
| 7-Jun | 0 | 0 | 0 | 0 | 4 | 4 |
| 8-Jun | 0 | 0 | 0 | 0 | 3 | 3 |
| 9-Jun | 0 | 0 | 0 | 0 | 3 | 3 |
| 10-Jun | 0 | 0 | 0 | 0 | 11 | 11 |
| 11-Jun | 0 | 0 | 0 | 1 | 6 | 7 |
| 12-Jun | 0 | 0 | 0 | 6 | 5 | 11 |
| 13-Jun | 0 | 0 | 0 | 38 | 19 | 57 |
| 14-Jun | 0 | 0 | 0 | 39 | 10 | 49 |
| 15-Jun | 0 | 0 | 0 | 32 | 8 | 40 |
| 16-Jun | 0 | 0 | 0 | 33 | 11 | 44 |
| 17-Jun | 0 | 0 | 0 | 21 | 4 | 25 |
| 18-Jun | 0 | 0 | 0 | 28 | 4 | 32 |
| 19-Jun | 0 | 0 | 0 | 23 | 4 | 27 |
| 20-Jun | 0 | 0 | 0 | 25 | 1 | 26 |
| 21-Jun | 0 | 0 | 0 | 11 | 7 | 18 |
| 22-Jun | 0 | 0 | 0 | 13 | 9 | 22 |
| 23-Jun | 0 | 0 | 0 | 9 | 3 | 12 |
| 24-Jun | 0 | 0 | 0 | 14 | 5 | 19 |
| 25-Jun | 0 | 0 | 0 | 11 | 2 | 13 |
| 26-Jun | 0 | 0 | 0 | 17 | 4 | 21 |
| 27-Jun | 0 | 0 | 0 | 14 | 3 | 17 |
| 28-Jun | 0 | 0 | 0 | 7 | 5 | 12 |
| 29-Jun | 0 | 0 | 0 | 8 | 11 | 19 |
| 30-Jun | 0 | 0 | 0 | 3 | 0 | 3 |

Note: The data were obtained from the Shenzhen Municipal Health Commission, and National Health Commission of the People’s Republic of China.
