# Supplementary material for: Changes in Compliance With Personal Preventive Measures and Mental Health Status Among Chinese Factory Workers During the COVID-19 Pandemic: An Observational Prospective Cohort Study
Source: Front Public Health. 2022 Mar 10;10:831456. doi: 10.3389/fpubh.2022.831456 (PMC8960195; doi:10.3389/fpubh.2022.831456)
Supplement: Supplementary file 2 [file Table_2.DOCX]

**Appendix 2 Chinese and English versions of the baseline and follow-up questionnaire**

**Baseline survey**

**基线问卷**

1 Frequency of facemask wearing in public spaces/transportations other than workplaces in the past month

过去一个月内，您在公众场所或者搭乘公共交通工具时是否佩戴口罩?

□_1_ Every time 每次都佩戴 □_2_ Often 经常佩戴 □_3_ Sometimes有时佩戴 □_4_ Never 从不佩戴

2 Frequency of facemask wearing when you have close contact with other people in workplace in the past month

过去一个月内，您上班与他人有近距离接触的时候是否佩戴口罩？

□_1_ Every time 每次都佩戴 □_2_ Often 经常佩戴 □_3_ Sometimes有时佩戴 □_4_ Never 从不佩戴

3 Types of facemask did you use in the past month (you may choose more than one responses)

过去一个月内，您所佩戴的口罩是否包括以下的类型? (可多选)

□_1_ Surgical mask 外科口罩

□_2_ Non-surgical grade respirators普通医用口罩

□_3_ N-95 mask N95口罩

□_4_ Cloth mask布口罩

□_5_ Did not use facemask in the past month过去一个月没有佩戴口罩

4 Whether you have reused face mask in the past month?

过去一个月内，你有没有反复使用之前用过的口罩?

□_1_ Yes 有 □_2_ No 没有

□_3_ Did not use facemask in the past month 过去一个月没有佩戴口罩

5 Frequency of sanitizing hands (using soaps, liquid soaps or alcohol-based sanitizer) after returning from public spaces or touching public installation in the past month

过去一个月内，你去过公共地方或接触公共设施后有没有用肥皂/洗手液洗手或者消毒双手？

□_1_ Every time 每次都用 □_2_ Often 经常用 □_3_ Sometimes 有时用 □_4_ Never 从不

6 Frequency of household disinfection in the past month

过去一个月内，你是否经常消毒家居

□_1_ Always 经常 □_2_ Sometimes 有时 □_3_ Seldom 很少 □_4_ Never 从不

| 7 In the past month, did you ……  在过去一个月内，你有没有…… | Yes | No |
| --- | --- | --- |
| A. Avoid social/meal gathering with other people who do not live together  避免与非同住的人聚会/聚餐 | 1 | 2 |
| B. Avoid crowded places  避免到人多的地方 | 1 | 2 |

8 How often have you been bothered by the following over the past two weeks?

在过去两个星期，有多少时候您受到以下任何问题所困扰

|  | **Not at all** | **Several days** | **More than half the days** | **Nearly every day** |
| --- | --- | --- | --- | --- |
| A. Little interest or pleasure in doing things?  做事时提不起来劲或没有乐趣 | 0 | 1 | 2 | 3 |
| B. Feeling down, depressed, or hopeless?  感到心情低落、沮丧或绝望 | 0 | 1 | 2 | 3 |
| C. Trouble falling or staying asleep, or sleeping too much?  入睡困难、睡不安稳或睡眠过多 | 0 | 1 | 2 | 3 |
| D. Feeling tired or having little energy?  感觉疲倦或没有活力 | 0 | 1 | 2 | 3 |
| E. Poor appetite or overeating?  食欲不振或吃太多 | 0 | 1 | 2 | 3 |
| F. Feeling bad about yourself --- or that you are a failure or have let yourself or your family down?  觉得自己很糟，或觉得自己很失败，或让自己或家人失望 | 0 | 1 | 2 | 3 |
| G. Trouble concentrating on things, such as reading the newspaper or watching television?  很难集中精力做一件事，比如读报或者看电视 | 0 | 1 | 2 | 3 |
| H. Moving or speaking so slowly that other people could have noticed? Or so fidgety or restless that you have been moving a lot more than usual?  感觉比平常更烦躁、坐立不安、动来动去 | 0 | 1 | 2 | 3 |
| I. Thoughts that you would be better off dead, or thoughts of hurting yourself in some way?  有不如死掉或用某种方法伤害自己的念头 | 0 | 1 | 2 | 3 |

9 During the past 7 days, how would you rate your sleep quality overall? (Please mark only 1 box from 0-10)

最后，我们想请您给过去一周的总体睡眠质量打个分（0分代表非常差，10分代表非常好，请您根据自己的感觉选择0-10分之间的一个分数）

| Terrible  非常差 |  | Poor  差 |  |  | Fair  一般 |  |  | Good  好 |  | Excellent  非常好 |
| --- | --- | --- | --- | --- | --- | --- | --- | --- | --- | --- |
| 0 | 1 | 2 | 3 | 4 | 5 | 6 | 7 | 8 | 9 | 10 |

10 How old are you: _____ years

您的年龄是：_______岁

11 What is your gender? □_1_Male □_2_ Female

您的性别为：□_1_男 □_2_ 女

12 Where are you come from? ______________

您的家乡在哪里: _______

13 What is your relationship status?

请问您目前的婚姻状况是：

□_1_ Without a stable boyfriend/girlfriend 未婚且没有固定的男/女朋友

□_2_ With a stable boyfriend/girlfriend 未婚但是有固定的男/女朋友

□_3_ Married 已婚

□_4_ Divorced/widowed 离婚或丧偶

14 What is your education level?

请问您的学历是：

□_1_ Primary school or below 小学或以下

□_2_ Junior high 初中

□_3_ Senior high or equivalent 高中或中专

□_4_ College 大专

□_5_ University 大学

□_6_ Postgraduate 研究生

15 What is your monthly income level?

请问你的平均月收入是多少

□_1_ Below 1000RMB

□_2_ 1000-2999 RMB

□_3_ 3000-4999 RMB

□_4_ 5000-6999 RMB

□_5_ 7000-9999 RMB

□_6_ 10,000 RMB or above

16 Are you a frontline worker or a management staff?

请问你是前线工人还是管理层

□_1_ Frontline worker 前线工人 □_2_ Management staff 管理层

**End of questionnaire**

**问卷结束**

**Month 3 follow-up survey**

**第三个月随访问卷**

1 Frequency of facemask wearing in public spaces/transportations other than workplaces in the past month

过去一个月内，您在公众场所或者搭乘公共交通工具时是否佩戴口罩?

□_1_ Every time 每次都佩戴 □_2_ Often 经常佩戴 □_3_ Sometimes有时佩戴 □_4_ Never 从不佩戴

2 Frequency of facemask wearing when you have close contact with other people in workplace in the past month

过去一个月内，您上班与他人有近距离接触的时候是否佩戴口罩？

□_1_ Every time 每次都佩戴 □_2_ Often 经常佩戴 □_3_ Sometimes有时佩戴 □_4_ Never 从不佩戴

3 Types of facemask did you use in the past month (you may choose more than one responses)

过去一个月内，您所佩戴的口罩是否包括以下的类型? (可多选)

□_1_ Surgical mask 外科口罩

□_2_ Non-surgical grade respirators普通医用口罩

□_3_ N-95 mask N95口罩

□_4_ Cloth mask布口罩

□_5_ Did not use facemask in the past month过去一个月没有佩戴口罩

4 Whether you have reused face mask in the past month?

过去一个月内，你有没有反复使用之前用过的口罩?

□_1_ Yes 有 □_2_ No 没有

□_3_ Did not use facemask in the past month 过去一个月没有佩戴口罩

5 Frequency of sanitizing hands (using soaps, liquid soaps or alcohol-based sanitizer) after returning from public spaces or touching public installation in the past month

过去一个月内，你去过公共地方或接触公共设施后有没有用肥皂/洗手液洗手或者消毒双手？

□_1_ Every time 每次都用 □_2_ Often 经常用 □_3_ Sometimes 有时用 □_4_ Never 从不

6 Frequency of household disinfection in the past month

过去一个月内，你是否经常消毒家居

□_1_ Always 经常 □_2_ Sometimes 有时 □_3_ Seldom 很少 □_4_ Never 从不

| 7 In the past month, did you ……  在过去一个月内，你有没有…… | Yes | No |
| --- | --- | --- |
| A. Avoid social/meal gathering with other people who do not live together  避免与非同住的人聚会/聚餐 | 1 | 2 |
| B. Avoid crowded places  避免到人多的地方 | 1 | 2 |

8 How often have you been bothered by the following over the past two weeks?

在过去两个星期，有多少时候您受到以下任何问题所困扰

|  | **Not at all** | **Several days** | **More than half the days** | **Nearly every day** |
| --- | --- | --- | --- | --- |
| A. Little interest or pleasure in doing things?  做事时提不起来劲或没有乐趣 | 0 | 1 | 2 | 3 |
| B. Feeling down, depressed, or hopeless?  感到心情低落、沮丧或绝望 | 0 | 1 | 2 | 3 |
| C. Trouble falling or staying asleep, or sleeping too much?  入睡困难、睡不安稳或睡眠过多 | 0 | 1 | 2 | 3 |
| D. Feeling tired or having little energy?  感觉疲倦或没有活力 | 0 | 1 | 2 | 3 |
| E. Poor appetite or overeating?  食欲不振或吃太多 | 0 | 1 | 2 | 3 |
| F. Feeling bad about yourself --- or that you are a failure or have let yourself or your family down?  觉得自己很糟，或觉得自己很失败，或让自己或家人失望 | 0 | 1 | 2 | 3 |
| G. Trouble concentrating on things, such as reading the newspaper or watching television?  很难集中精力做一件事，比如读报或者看电视 | 0 | 1 | 2 | 3 |
| H. Moving or speaking so slowly that other people could have noticed? Or so fidgety or restless that you have been moving a lot more than usual?  感觉比平常更烦躁、坐立不安、动来动去 | 0 | 1 | 2 | 3 |
| I. Thoughts that you would be better off dead, or thoughts of hurting yourself in some way?  有不如死掉或用某种方法伤害自己的念头 | 0 | 1 | 2 | 3 |

9 During the past 7 days, how would you rate your sleep quality overall? (Please mark only 1 box from 0-10)

最后，我们想请您给过去一周的总体睡眠质量打个分（0分代表非常差，10分代表非常好，请您根据自己的感觉选择0-10分之间的一个分数）

| Terrible  非常差 |  | Poor  差 |  |  | Fair  一般 |  |  | Good  好 |  | Excellent  非常好 |
| --- | --- | --- | --- | --- | --- | --- | --- | --- | --- | --- |
| 0 | 1 | 2 | 3 | 4 | 5 | 6 | 7 | 8 | 9 | 10 |

**Thank you for your support!**

**问卷结束，谢谢你的支持**
