# Supplementary material for: Changes in Compliance With Personal Preventive Measures and Mental Health Status Among Chinese Factory Workers During the COVID-19 Pandemic: An Observational Prospective Cohort Study
Source: Front Public Health. 2022 Mar 10;10:831456. doi: 10.3389/fpubh.2022.831456 (PMC8960195; doi:10.3389/fpubh.2022.831456)
Supplement: Supplementary file 3 [file Table_3.DOCX]

Appendix 3 Comparing depressive symptoms and sleep quality measured at Month 3 among subgroups of participants with different compliance with personal preventive measures at baseline and Month 3 follow-up

|  | Depressive symptoms at Month 3 | | | Sleep quality at Month 3 | | |
| --- | --- | --- | --- | --- | --- | --- |
| Subgroup | Mean (SD) | B (95%CI) | P  values | Mean (SD) | B (95%CI) | P values |
| Compliance to facemask wearing in workplace |  |  |  |  |  |  |
| Both baseline and Month 3 (n=585) | 0.6 (1.6) | Ref | Ref | 8.4 (1.4) | Ref | Ref |
| Baseline only (n=65) | 1.6 (3.4) | 0.11 (-0.01, 0.24) | 0.08 | 7.8 (1.6) | -0.05 (-0.07, -0.02) | <0.001 |
| Month 3 only (n=9) | 1.3 (1.8) | 0.14 (-0.17, 0.45) | 0.38 | 8.2 (1.2) | -0.01 (-0.07, 0.05) | 0.80 |
| Neither baseline nor Month 3 (n=4) | 0 (N.A.) | N.A. | N.A. | 7.8 (1.0) | -0.03 (-0.12, 0.06) | 0.49 |
| Compliance to facemask wearing in other public spaces |  |  |  |  |  |  |
| Both baseline and Month 3 (n=595) | 0.6 (1.8) | Ref | Ref | 8.4 (1.4) | Ref | Ref |
| Baseline only (n=49) | 1.0 (2.4) | 0.10 (-0.07, 0.27) | 0.25 | 8.1 (1.9) | -0.03 (-0.05, 0.001) | 0.06 |
| Month 3 only (n=18) | 0.5 (1.0) | 0.05 (-0.26, 0.36) | 0.75 | 8.7 (1.6) | 0.02 (-0.03, 0.06) | 0.49 |
| Neither baseline nor Month 3 (n=1) | 0 (N.A.) | N.A. | N.A. | 10 (N.A.) | 0.08 (-0.10, 0.27) | 0.37 |
| Sanitizing hands after returning from public spaces or touching public installation |  |  |  |  |  |  |
| Both baseline and Month 3 (n=255) | 0.5 (1.4) | Ref | Ref | 8.4 (1.5) | Ref | Ref |
| Baseline only (n=215) | 0.7 (1.5) | -0.09 (-0.21, 0.03) | 0.14 | 8.4 (1.4) | -0.002 (-0.02, 0.02) | 0.80 |
| Month 3 only (n=61) | 0.9 (2.3) | 0.12 (-0.06, 0.31) | 0.20 | 8.5 (1.4) | 0.007 (-0.02, 0.03) | 0.62 |
| Neither baseline nor Month 3 (n=132) | 0.7 (1.9) | 0.01 (-0.12, 0.13) | 0.92 | 8.2 (1.5) | -0.01 (-0.03, 0.01) | 0.26 |
| Avoid social/meal gathering with other people who do not live together |  |  |  |  |  |  |
| Both baseline and Month 3 (n=409) | 0.7 (1.6) | Ref | Ref | 8.3 (1.5) | Ref | Ref |
| Baseline only (n=101) | 0.5 (1.2) | -0.01 (-0.15, 0.14) | 0.92 | 8.1 (1.5) | -0.01 (-0.03, 0.01) | 0.30 |
| Month 3 only (n=116) | 0.8 (2.9) | 0.10 (-0.04, 0.24) | 0.16 | 8.5 (1.3) | 0.01 (-0.07, 0.03) | 0.20 |
| Neither baseline nor Month 3 (n=37) | 0.4 (1.3) | -0.002 (-0.28, 0.28) | 0.99 | 8.7 (1.4) | 0.02 (-0.01, 0.05) | 0.18 |
| Avoid crowded places |  |  |  |  |  |  |
| Both baseline and Month 3 (n=363) | 0.6 (1.6) | Ref | Ref | 8.4 (1.4) | Ref | Ref |
| Baseline only (n=100) | 0.5 (1.3) | 0.01 (-0.15, 0.16) | 0.99 | 8.2 (1.5) | -0.01 (-0.03, 0.01) | 0.36 |
| Month 3 only (n=148) | 0.8 (2.7) | 0.07 (-0.05, 0.20) | 0.23 | 8.4 (1.5) | 0.01 (-0.01, 0.03) | 0.42 |
| Neither baseline nor Month 3 (n=52) | 0.6 (2.0) | -0.01 (-0.20, 0.18) | 0.89 | 8.2 (1.3) | 0.01 (-0.02, 0.05) | 0.53 |
| Household disinfection |  |  |  |  |  |  |
| Both baseline and Month 3 (n=149) | 0.5 (1.8) | Ref | Ref | 8.5 (1.7) | Ref | Ref |
| Baseline only (n=161) | 0.6 (1.3) | -0.08 (-0.24, 0.07) | 0.31 | 8.5 (1.3) | 0.007 (-0.01, 0.03) | 0.49 |
| Month 3 only (n=106) | 0.5 (1.4) | -0.05 (-0.23, 0.13) | 0.62 | 8.4 (1.3) | 0.001 (-0.02, 0.03) | 0.91 |
| Neither baseline nor Month 3 (n=247) | 0.8 (2.3) | -0.01 (-0.16, 0.13) | 0.87 | 8.2 (1.4) | -0.007 (-0.03, 0.01) | 0.46 |

B: unstandardized coefficients obtained from multilevel linear regression models (level 1: factories, level 2: individual participants)

Score of PHQ-9 and the single-item Sleep Quality Scale were log transformed using the base of 10 and were used as dependent variables in the multilevel linear regression models.
